# Immobilized Nanomaterials for Environmental Applications

**DOI:** 10.3390/molecules27196659

**Published:** 2022-10-07

**Authors:** Francisco J. Cervantes, Luis A. Ramírez-Montoya

**Affiliations:** Laboratory for Research on Advanced Processes for Water Treatment, Engineering Institute, Campus Juriquilla, Universidad Nacional Autónoma de México (UNAM), Blvd. Juriquilla 3001, Querétaro 76230, Mexico

**Keywords:** biodegradation, bioenergy production, greenhouse gases, industrial wastewater treatment, recalcitrant contaminants

## Abstract

Nanomaterials (NMs) have been extensively used in several environmental applications; however, their widespread dissemination at full scale is hindered by difficulties keeping them active in engineered systems. Thus, several strategies to immobilize NMs for their environmental utilization have been established and are described in the present review, emphasizing their role in the production of renewable energies, the removal of priority pollutants, as well as greenhouse gases, from industrial streams, by both biological and physicochemical processes. The challenges to optimize the application of immobilized NMs and the relevant research topics to consider in future research are also presented to encourage the scientific community to respond to current needs.

## 1. Introduction

**Figure 1:**
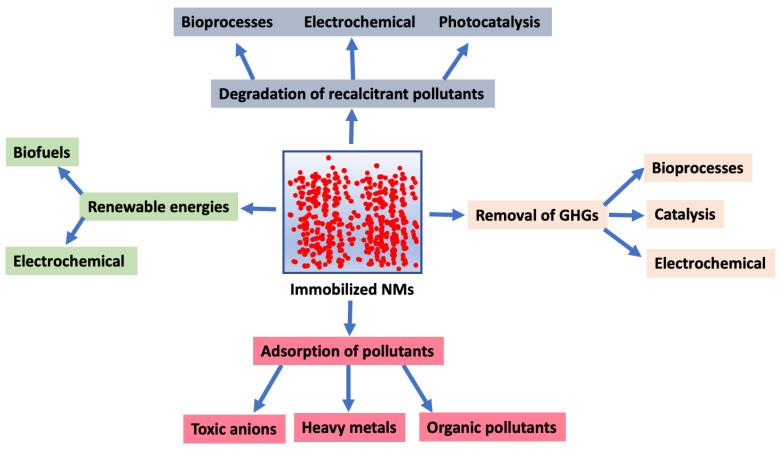
Key environmental applications of immobilized nanomaterials (NMs). GHGs, greenhouse gases.

The superior catalytic properties of nanomaterials (NMs) as compared to their bulk precursors have encouraged their application in several fields, including for environmental purposes, such as the removal of persistent contaminants, stimulation of microbial processes for bioenergy production, and reduction of greenhouse gas (GHG) emissions [1,2]. Nevertheless, NMs cannot be used in suspension in engineered systems since their recovery is expensive and unreasonable in post-treatments in full-scale operations. Furthermore, the discharge of NMs to environmental reservoirs represents a serious risk, and their use in engineered systems could be counterproductive, since many of these manufactured materials may have environmental and public health consequences, such as genotoxicity and cytotoxicity [3]. Accordingly, strategies to immobilize NMs in treatment systems for several distinct applications have been developed. These immobilizing techniques are particularly relevant for environmental purposes. Among the main advantages related to the use of immobilized NMs as catalysts are: (1) facile separation and retention of the catalysts in the reaction system; (2) continuous operation proceeds smoothly; and (3) the combined effects of catalysis and adsorption with NMs cover both commitments. Nevertheless, some disadvantages could also arise from the use of immobilized NMs which need to be addressed in future works, such as deactivation, poisoning, or washout of the catalysts, as well as mass-transfer limitations [4]. The purpose of this review is to provide an overview of the main strategies to immobilize NMs for their application in different environmental engineered processes (Figure 1). Particularly, this paper summarizes the application of immobilized NMs in the production of renewable energies, as well as the removal of recalcitrant pollutants and GHGs from industrial discharges.

## 2. Immobilized NMs for Energy Production

### 2.1. Biofuels

Global warming caused by anthropogenic emissions associated with the conventional production of fuels has exacerbated the living conditions of our planet, putting at risk the sustainability required for future generations. Consequently, the introduction of renewable energy sources to replace fossil fuels will play a pivotal role in tackling this menace. Methane is a renewable energy source, which can be sustainably obtained in engineered systems during the treatment of industrial wastewaters, as well as solid residues, such as animal manure or the organic fraction of municipal solid waste, by a process denominated anaerobic digestion (AD) [5]. AD comprises a complex route in which several anaerobic microorganisms are responsible for converting organic substrates into biogas under anaerobic conditions. In the last few years, the role of conductive NMs on AD processes has received a lot of attention because their application to anaerobic bioreactors has significantly increased the methane production from synthetic and real wastewaters [5]. NMs applied in these studies include carbon-based materials, such as graphene oxide (GO) and carbon nanotubes (CNT), in addition to metallic NMs, such as magnetite, nano zero-valent iron (nZVI), silver, and different iron oxides [6].

Recently, direct interspecies electron transfer (DIET) has been reported involving the formation of an electric current between electron-donating and electron-accepting microorganisms. For example, DIET has been shown to occur in cocultures of *Geobacter* species and acetoclastic methanogens, such as *Methanosarcina* sp., and this electron-transferring mechanism has been pointed out as one of the reasons for the improved performance of anaerobic bioreactors for producing methane in the presence of conductive materials (CM) [7]. Certainly, it was shown that the lack of pili and other cellular structures involved in electron transfer in microbial consortia can be compensated by the presence of CM [7,8]. Nevertheless, additional studies have indicated that the input of CM on AD processes goes beyond the stimulation of DIET because electric conductivity is not the only relevant parameter [6,9]. In fact, several physical-chemical properties of NMs, such as particle size, functional group, and surface area, drive the interactions between these materials and anaerobic microorganisms, ultimately determining the performance of anaerobic bioreactors during the production of methane from wastewater and solid residues. For instance, oxidized functional groups in GO promoted strong interaction between this material and anaerobic microorganisms, in a methanogenic consortium, causing the wrapping of cells and, consequently triggering mass-transfer limitations during the conversion of complex substrates, such as starch, which was reflected in a lower production of biogas [10]. However, when GO was applied in its reduced form (GOr) to the same consortium, this CM did not cover the cells and stimulated a greater production of methane [11].

Efforts to apply immobilized NMs to improve the production of methane from wastewater have recently been reported. For instance, nano-magnetite was fixed in granular activated carbon (GAC) for its subsequent application for the anaerobic treatment of low-strength wastewater [12]. The synthesized composite was mixed with anaerobic sludge in a bioreactor, which showed superior conductivity and electron-transferring capacity, ultimately fueling 3.6-fold higher methane production, as compared to the control bioreactor. Interestingly, the application of immobilized nano-magnetite triggered a higher abundance of functional microorganisms. Additionally, a strategy for in situ formation and self-immobilization of biogenic iron oxides in anaerobic granular sludge revealed an effective improvement of the production of methane, which was attributed to increased conductivity and stimulated growth of exoelectrogens (i.e., microorganisms with the capacity to donate electrons, such as *Clostridium*) and endoelectrogens (i.e., microorganisms with the ability to accept electrons, such as *Methanosaeta*) [13]. More recently, GO was immobilized in organic xerogels, which were applied for the treatment of protein-rich wastewater. The addition of this composite promoted higher methane production and the superior removal of organic matter and ammonium, as well as the production of medium chain fatty acids [14].

Hydrogen (H_2_) is the only carbon-free fuel available, and is expected to play a crucial role in the future energy market due to its high energy content and clean combustion product [15]. H_2_ can also be obtained by anaerobic consortia under more controlled conditions (dark fermentation). Furthermore, several metallic NMs, such as nanoparticles (NPs) of Ag, Au, Ni, and Fe_2_O_3_, have been shown to enhance H_2_ production in terms of production rate, as well as yield [16,17,18,19]; thus, it is conceivable that these NMs could also be immobilized and applied in dark fermentation processes for improving the production of this green biofuel. Besides this, the immobilization of *Enterobacter aerogenes* on CNT improved the H_2_ production rate and yield as compared to the performance observed with free cells [20].

Magnetic nanoparticles (MNPs) represent another efficient way to keep catalysts immobilized in bioreactors, and has successfully been applied for improving AD processes [6]. Besides this, MNPs have also been extensively explored as a carrier material for immobilizing enzymes linked to biodiesel and bioethanol production [21]. Immobilized microorganisms/enzymes in MNPs can easily be recovered from fermentation broth for subsequent use in several cycles, which significantly decreases the operational costs in biofuel production. For example, immobilized *Saccharomyces cerevisiae* achieved an ethanol productivity of 264 g/L-h from corn starch and was effectively maintained in several cycles for more than a month [22]. Furthermore, cellulase immobilized in MNPs achieved an efficient hydrolysis of *Sesbania aculeate* biomass yielding up to 5.3 g/L of ethanol along with the reuse of the nano-biocatalysts several times [23]. In another study, biodiesel production from microalgal oil was sustained for up to seven regenerations with a yield reaching 94% under optimal conditions [24]. 

### 2.2. Electrochemical Energy Applications

Biogenic NPs have been integrated into bio-electrochemical systems, together with different microorganisms to produce biohydrogen or bioelectricity. For instance, Orozco et al. (2010) [25] combined Pd NPs with *E. coli* for the conversion of H_2_ into energy in a fuel cell. Similar studies were also reported in bio-electrochemical systems in which Pd or Pt NPs were immobilized, together with cultures of *Pseudomonas putida* [26], *Desulfovidrio desulfuricans* [27], *Shewanella oneidensis* [28], and *Saccharomyces cerevisiae* [29].

Graphene, jointly immobilized with NPs, has also appeared as a stirring material applied in electrochemical systems for energy storage and conversion applications in lithium batteries and supercapacitors, among other power applications [30]. In fact, the manufacturing of composites created with metallic NPs and graphene has allowed us to overcome some limitations during the application of these NMs in lithium batteries (LBs). For example, Co_3_S_4_ has the potential to be used as anode material in LBs, but shows capacity vanishing, low conductivity, and poor cyclability [31]. In contrast, the deposition of Co_3_S_4_ nanotubes on graphene allows better cycling performance and greater reversible capacity as compared to the pristine Co_3_S_4_ electrode [32]. The additional doping of anodes with elements belonging to group IV, such as Ge and Sn, has significantly enhanced Li-storage capacities, conductivity, reversible capacity, coulombic efficiency, and rate capability [30].

Supercapacitors (SCs) are electrochemical power-storage devices, which compile and discharge energy by reversible adsorption and desorption of ions at the boundaries between electrode constituents and electrolytes. Due to its large available surface, graphene represents an attractive material for electrodes in SCs [33]. In addition, the doping of graphene with Ni(OH)_2_ NPs resulted in better electrode–electrolyte interaction, enhancing the conductivity of ions and electrons, ultimately resulting in a superior performance of SCs [34].

Further applications of immobilized NMs in electrochemical energy devices include their employment as composite catalysts to convert solar energy into chemical energy by splitting water into hydrogen photocatalytically. Additionally, graphene-based composites have successfully been applied as photocatalysts to reduce CO_2_ into reactive carbon forms [30]. Table 1 summarizes the application of immobilized NMs for energy production.

## 3. Immobilized NMs for the Removal of Recalcitrant Contaminants

### 3.1. Bioprocesses

**Figure 2:**
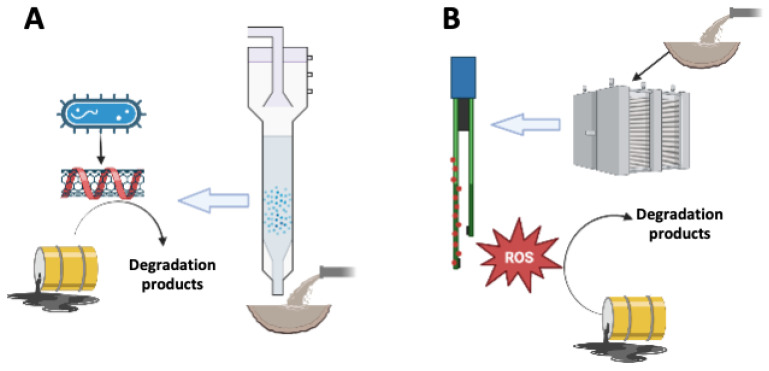
Mechanisms involved in the degradation of recalcitrant pollutants mediated by immobilized NMs in bioprocesses (**A**) and in electrochemical systems (**B**). Bacteria reduce conductive NMs (e.g., nanotubes), which promote redox reactions driving the biodegradation of contaminants (**A**). Immobilized NMs in electrochemical systems produce reactive oxygen species (ROS), which stimulate the degradation of contaminants (**B**).

Recalcitrant pollutants are defined as those in which the chemical structure is very stable and, consequently, are hardly degraded in conventional biological treatment systems. Many of these recalcitrant compounds are found at trace levels in wastewater; yet, due to their high environmental risks associated with human health and ecotoxicology, their removal is a priority [37]. In order to increase the biodegradability of persistent contaminants, distinct nano-catalysts have been applied in bioreactors. Particularly, strategies to immobilize these NMs to enhance the biodegradation of recalcitrant pollutants have recently been reported (Figure 2). For instance, De Corte et al. (2012) [38] proposed several retention mechanisms for applying biogenic nano-palladium (Bio-Pd) in the bioremediation of waters contaminated with chlorinated solvents and pharmaceuticals in different reactor configurations, such as retention by hollow fiber and plate membranes, encapsulation in alginate beads and polymeric membranes, and coating on zeolites. Bio-Pd has also been immobilized in anaerobic granular sludge for its application as a biocatalyst in the anaerobic biotransformation of pharmaceuticals, chlorinated solvents, azo dyes, nitro-aromatics, and Cr^6+^ [37,39,40]. Additionally, Bio-Pd has been immobilized in the cathode of a bio-electrochemical reactor, which achieved a removal rate of tri-chloro-ethylene (TCE) of 151 g/m^3^-d. This removal rate allowed 93% removal of TCE in the Pd-doped system, while only 48% removal was observed in the absence of this nano-catalyst [41].

Furthermore, graphene-anaerobic sludge composite was shown to be effective for enhancing the rate of reductive biotransformation of nitrobenzene up to twofold [42]. More recently, magnetic graphene oxide nano-sacs (MGONS), immobilized in anaerobic bioreactors, greatly stimulated the biotransformation of iopromide. Indeed, a higher removal efficiency and greater extent of biodegradation occurred in the bioreactor amended with MGONS as compared to the control system lacking this catalyst [43]. NMs have also served as a core for holding redox-active molecules, such as quinones and humic substances, and their application has greatly enhanced the reductive biotransformation of chlorinated solvents and azo dyes [44,45,46].

Additional applications were reported with microbial cells of *Pseudomonas delafieldii* coated with MNPs, which improved the mass-transfer rate during the desulfurization of dibenzothiophene, which could be relevant for engineered treatment systems in the petrochemical sector [47].

### 3.2. Electrochemical Systems

Electrochemical technologies, such as electro-oxidation, electro-coagulation, and electro-flotation, have gained attention for the treatment of complex wastewater, disinfection of drinking water, as well as the removal of hazardous, persistent contaminants [48]. Electro-oxidation has emerged as an environmentally friendly process remediation technique, since few or no chemicals are needed to facilitate wastewater treatment, reaching more efficient means, low energy consumption, and convenient operation during wastewater treatment as compared to the biological (aerobic and anaerobic) and physicochemical (membrane technologies, adsorption, and ozonation) alternatives [49]. Electro-oxidation involves the production of strong oxidants (particularly hydroxyl radicals) during the treatment in situ, either directly at the electrode or indirectly from chemical compounds in the treated water [50]. Among the advanced oxidation processes (AOPs), electro-Fenton is an effective method to degrade refractory organic compounds, including dyes, phenol compounds, oil-refining wastewater, and pharmaceutical compounds [51,52,53]. However, several drawbacks, such as particle agglomeration, low recyclability, and high dependence on the main electrode, still exist, hindering its application [54]. The use of heterogenous nanocomposites appears an alternative to overcome these disadvantages, since the development of stable, inexpensive, selective, and high-performing electrocatalysts is imperative to scaling up this technology to industrial levels. Recently, the immobilization of bimetallic NPs (Ni/Co) on metal organic frameworks (MOF), based on nitrogen-doped porous carbon rods, was carried out for the development of an efficient heterogenous electro-Fenton catalyst and successfully used in the degradation of organic compounds, including tetracycline, chloramphenicol, ciprofloxacin, diclofenac sodium, and sulfamethoxazole, due to reactive oxygen species (ROS) driving the reactions [55]. MOF were also used for the encapsulation of Fe_3_O_4_ NPs, showing an enhanced degradation of heterocycles, phenols, and esters in old landfill leachate [56]. Changes in the electrode are also reported for the electro-oxidation of diclofenac by the tailoring of a Cu-GOr electrode with a high oxidative current response and high removal efficiencies [57]. Moreover, the electrocatalytic degradation of methyl orange was accomplished using nano-Fe_3_O_4_ supported on carbon black, which was used as electrode [49].

### 3.3. Photocatalytic Applications

Photocatalytic degradation of recalcitrant pollutants, using nano-catalysts, has gained attention due to its efficiency, cost effectiveness, low toxicity, low harmfulness, high durability, super-hydrophilicity, photochemical and chemical stability features, and eco-friendly status, leading to complete mineralization and the overcoming of the drawbacks of traditional techniques [58]. The entire mineralization of organic compounds is reached when a semiconductor is irradiated with some light source promoting the formation of valence band holes due to electron excitation, where reactive oxygen species are produced [59]. Photocatalysis is a type of AOP that relies on hydroxyl-radical generation, based on the interaction of a photocatalyst with the incident light energy. This technology is very mature and there are several reviews about its use [60,61], with an estimate of 48,000 publications about dye photocatalytic degradation only under UV and visible light irradiation [62]. Recent trends have led to the development of nano-photocatalysis, since the advantages of NMs result in greater photocatalytic activity and complete mineralization of some pollutants into simple compounds. In nano-catalysis, the movement of electrons and holes, which is crucial for the application of photocatalysis, is also affected by the size and configuration of the NMs.

However, nano-catalysts need to be immobilized in organic/inorganic matrixes to allow liquid–solid separation and recycling efficiency for an effective degradation process [63]. Several photocatalysts have been used for environmental applications (TiO_2_, ZnO, WO_3_, CuS, SnO_2_, CdS, etc.), but most of these photocatalysts are only active under UV light because of a comparatively large bandgap. In terms of supporting materials, the most commonly used include glass, stainless steel, plastics, textiles, alumina, silica, and titania [64]. Recent works in this area are focused on the generation of low-cost nanocatalysts using wasted and recyclable materials as carriers for applications in the removal of different target contaminants, such as dyes, pharmaceutical compounds, and heavy metals [65]. Materials such as polyethylene terephthalic (PET), polystyrene (PS), disposal textile fabrics, newspapers, aluminum soda cans, rubber, waste electronic and electric components and used batteries have been employed as supports for immobilizing catalysts [65]. The use of these carriers represents an eco-friendly alternative for photocatalytic purposes. For instance, the use of wasted silicon powder for the immobilization of synthetic La/TiO_2_ NPs significantly improved the degradation of dimethyl phthalate [66]. Additionally, the potential of chitosan-conjugated manganese to produce a magnetic nano bio-composite for the treatment of emerging dye pollutants [58], and harnessing solar light using nano-ZnO supported on activated carbon (AC) for dye degradation [63] has been explored. Table 2 summarizes the application of immobilized NMs for the removal of recalcitrant pollutants.

## 4. Immobilized NMs for the Removal of GHGs

Technologies to remove GHGs from industrial processes, such as fossil fuel extraction, as well as from wastewater treatment and landfill facilities, are currently mandatory to decrease these anthropogenic emanations, which are contributing to the overall warming of our planet. In the following sections, the role of immobilized NMs on the removal of the three main GHGs (methane, nitrous oxide, and carbon dioxide) derived from human activities will be discussed.

### 4.1. Methane

While biogenic methane, produced from waste treatment, is considered a renewable energy source [14], there are important discharges of this gas during the exploitation of fossil energies. Additionally, methane is also released from wastewater-treatment plants and landfills. Methane is a powerful GHG with a global warming potential (GWP) 30-fold higher than that of carbon dioxide (CO_2_); therefore, technologies to remove this gas from industrial emissions are demanded [67].

The catalytic oxidation of methane has been explored during the last decade and Pd-based catalysts have shown the highest activity for methane oxidation. Nevertheless, the application of these materials is still challenging due to poisoning by water and insufficient low-temperature performance. Recently, immobilized PdO_x_ on two-dimensional rafts showed efficient methane oxidation and water tolerance, creating a new venue of opportunities [68].

Metallic NPs have also been immobilized in electrochemical systems, which have been applied for the oxidation of methane to organic solvents, such as methanol, ethanol, and formate [69]. This strategy constitutes a green technology, turning a GHG into organic supplies required in several industrial sectors. NMs applied in electrochemical processes for this purpose include alloys composed of V, Pd, Au, Cu, Ru, and Ni, among others. Selectivity is still a challenge in these processes as the conversion efficiency largely varies from 30% up to 100% depending on the operational conditions and immobilized catalysts applied (Table 2). Lately, Pd-Pt NPs immobilized on ceria showed remarkable efficiency in completely oxidizing methane under wet conditions, which are relevant for automotive combustion; thus, these scientific findings could be the basis to install a technology to prevent methane emissions from vehicles [70].

Anaerobic methane oxidation by microorganisms using the anode of bio-electrochemical systems as a terminal electron acceptor has recently been reported [71,72,73]; thus, the doping of electrodes with catalytic metals could also enhance the anodic AOM activities previously observed, which also constitutes an interesting topic to study in future research for potential applications.

### 4.2. Nitrous Oxide (N_2_O)

N_2_O is a serious environmental threat causing the acidification of water reservoirs, ozone-layer depletion, and with a global warming potential (GWP) 300-fold higher than that of carbon dioxide (CO_2_). Its concentration in the atmosphere has been augmented 18% since the preindustrial level, mainly due to human activities, such as the extensive application of fertilizers and burning of fossil fuels. Consequently, European Union edicts aim at its 42% reduction by 2030, with respect to 2013 emission data [74].

Selective catalytic reduction is the most successful and widely applied technology to mitigate N_2_O emissions, where this GHG is removed by different reducing agents (e.g., hydrogen, ammonia, hydrocarbons) in the presence of metallic catalysts. Electrochemical processes have recently emerged as an encouraging option to reduce N_2_O. Furthermore, the role of catalytic metals on the reduction in N_2_O has also been explored with distinct alloys, containing Ir, Pd, Re, and La, among other elements, which showed promising efficiency [75,76,77,78].

More recently, the reduction of N_2_O on the surface of electrodes, doped with NMs, was shown to be an effective strategy to remove this GHG from gas emissions in electrochemical systems. For instance, the deposition of Os and Pt NPs to create a stable film on the surface of different electrode materials was accomplished, and the doped electrodes were effective for reducing N_2_O to dinitrogen gas (N_2_) [79]. Lately, the reduction of N_2_O to N_2_ was also achieved on the surface of a cathode enriched with La, Sr, Fe, and Co [74]. The mechanism involved in this electrochemical process was elucidated, showing that the catalytic rate returns to its open circuit value after polarization. The cathode was shown to remain stable during the operation; thus, it is proposed as a promising technology to remove N_2_O from industrial exhaust emissions.

Considering that carbon-based materials, such as humic substances [80] and biochar (BC) [81], have been shown to effectively promote the reduction of N_2_O by microbial communities, it is conceivable that carbonaceous NMs, such as GO, could also serve as effective materials, immobilized in biocathodes for the reduction of this GHG. Additionally, bio-Pd NPs have been applied in denitrifying processes for enhancing the reduction of nitrate to N_2_ without N_2_O accumulation, which could also serve as the basis for engineering biosystems preventing N_2_O emissions from denitrifying processes [82]. Moreover, nZVI in the cathode of a bio-electrochemical system also enhances the reduction of nitrate without release of N_2_O [83].

### 4.3. Carbon Dioxide (CO_2_)

CO_2_ is the principal GHG responsible for global warming and it is mainly produced from power plants. While carbon-capture facilities contribute to decrease the release of CO_2_ from energy-generating infrastructure, the concentrated amines applied for this purpose are extremely toxic and difficult to treat after disposal [84]. Thus, green technologies are demanded to remove CO_2_ from gas emissions, including those derived from WWTP, for instance for upgrading biogas.

NMs immobilized by different fixing strategies have recently been used for removing CO_2_ from industrial gas streams. For example, nanocellulose-based membranes (NCM) have extensively been employed to eliminate this GHG. NCM offer great advantages for the removal of CO_2_, such as a high specific surface area, hydrophilicity, the option to modify their surface, environmental friendliness, and outstanding mechanical properties [85]. Since the diffusion mechanism involved in the separation of CO_2_ through NCM occurs very slowly, there are several challenges still to be addressed for promoting their extensive application in industrial processes. Moreover, since pH is a crucial parameter during the removal of CO_2_ in these processes, more studies are required for optimization. Additionally, NCM can also be doped with other NMs in order to enhance their performance, which will imply the optimization of membrane materials and tailoring of hybrid matrixes.

An exciting area of interest at present is the development of photo-bio-electrochemical systems, which involve the coupling between intact photosynthetic bacteria and electrodes, creating the proper niche to achieve “semi-artificial photosynthesis”, which may represent a new pathway for CO_2_ sequestration. The interfacing of photosynthetic bacteria with modified electrode surfaces and metallic NPs promotes enhanced current densities [86]. For instance, Gacitua et al. [87] developed an Os-based redox polymer, which was combined with the cyanobacterium, *Gloeocapsopsis* sp. UTEXB3054, achieving a photo-current density of 2.3 μA/cm^2^. Furthermore, Liu and Choi (2021) [88] reported the combination of biogenic Au NPs with *Synechocystis* sp. in a process which yielded 120 μA/cm^2^. An additional benefit of applying photo-bio-electrochemical systems for CO_2_ sequestration is the production of photosynthetic biomass with the proper oil content to produce biofuels. Therefore, there is a whole avenue opened for applying NMs in photo-bio-electrochemical systems, which may provide multiple environmental services.

## 5. Immobilized NMs for Adsorption Processes

**Figure 3:**
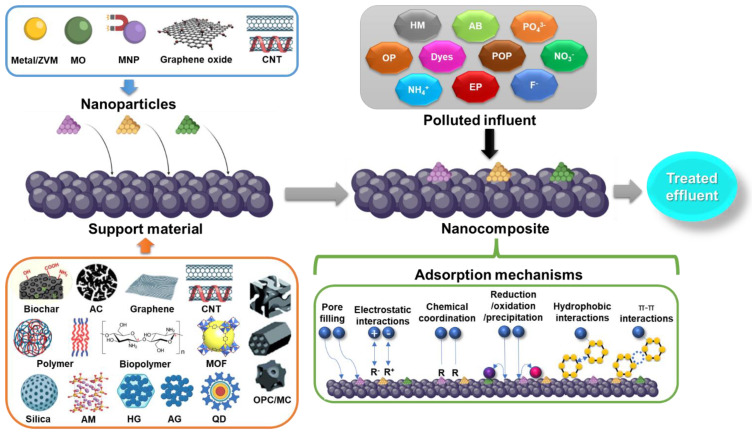
Scheme of nanoparticle immobilization on different supporting materials for the treatment of polluted discharges through adsorption processes. AB, antibiotic; AC, activated carbon; AG, aerogel; AM, alkali-metal-based material; CNT, carbon nanotube; EP, emerging pollutant; HG, hydrogel; HM, heavy metal; MC, monolithic carbon; MO, metal oxide; MOF, metal organic framework; MNP, magnetic nanoparticle; OP, organic pollutant; OPC, ordered porous carbon; POP, persistent organic pollutant; QD, quantum dot; ZVM, zero valent metal.

Adsorption processes are one of the technologies applied the most for the attenuation of environmental pollution. It is a traditional methodology based on the physicochemical interactions between the adsorbent/adsorbate that can be maximized using specific materials with a variety of tailored textural, mechanical, chemical, and electrical properties. Additionally, operational, economical, and environmental issues must be concerned during the selection and implementation of the adsorption system. Currently, the emergence of functional NMs and their innovative properties have gained attention as potential alternatives for environmental applications. The production of a broad range of nanocomposites has opened an unprecedent technological opportunity for a variety of applications, including adsorption, over the conventional use of these materials [89]. The use of immobilized NMs in adsorption processes includes the conjugation of NPs and support characteristics for a correct adsorptive performance. Many materials from different sources (carbon, silica, polymers) have been explored as NP carriers, including more innovative and sophisticated structures, such as graphene, biopolymers, CNT, MOF, aerogels and hydrogels, magnetic materials, quantum dots, and some hybrids integrating some of the previously mentioned materials [90,91,92,93,94,95]. Recently developed nanocomposites used for adsorption purposes are reviewed in this section with an emphasis on the type of immobilized NMs, host material, specific surface area (SSA), and adsorption capacity (Q) of different pollutants. Figure 3 schematizes the use of immobilized NMs for the treatment of polluted streams through adsorption processes.

### 5.1. Carbon-Based Nanocomposites

Their outstanding physical and chemical properties, coupled to their recognized versatility and stability under a variety of working conditions, make carbon-based materials an ideal option as a host material for NM immobilization. The large specific surface area, high pore volume, hydrophobic nature, high electrical conductivity, chemical inertness, and vast chemical functionality enables the loading of NPs through the carbonaceous matrix for successful applications in environmental remediation, energy storage, and catalysis [89,96,97]. AC is one of the most frequently used adsorbents for the removal of numerous pollutants from water and air bodies, mainly due to its high specific surface area, reaching values of 2878 m^2^/g for chemical activation with KOH and 2213 m^2^/g for steam activation [98], a characteristic that renders them suitable for the immobilization of a variety of NPs [99,100]. In the same way, BC has emerged as a low-cost alternative for metal NP immobilization [101], including the environmental and economic benefits associated with the use of biomass waste materials instead of non-renewable sources for AC production [102]. The intensive use of BC as a host material for NP immobilization is reflected in a recent review that summarized its performance as an nZVI support for heavy metal adsorption [103], and another focused on the preparation, environmental application, and prospect of BC-supported metal NPs [101]. Many sources for BC production, such as wood, coconut shell, corn straw, rice husk, nutshell, and peach stone, among others, have been explored, mainly based on their carbon content, abundance, inherent environmental issues, and mechanical properties [99,101,104]. Immobilization of NPs into a BC structure can enhance the advantages and overcome the drawbacks of both materials in terms of the distribution and stabilization of loaded metals, reducing the aggregation of NPs, their leaching and surface passivation, increasing the disposable active sites and oxygenated functional groups, and improving the catalytic/redox performance and their thermal stability [101,105,106].

Many pollutants have been targeted for remediation by adsorption using carbon-based nanocomposites. The removal of metal ions and dyes is one of the major areas of research for the use of porous materials, such as AC and BC, as well as carbon-based nanocomposites [107]. However, studies related to the removal of organic compounds (pesticides, antibiotics, and persistent organic pollutants), and inorganic species (phosphate, fluoride, ammonium, nitrate, etc.), have been recently reported for the treatment and remediation of water bodies and contaminated soils [83,100,108,109,110,111]. The properties of carbon materials used as supports are strongly affected by the integration of a variety of NPs, depending on the immobilization procedure (pre- or post-synthesis), modification treatment, and thermal processes applied. Physicochemical changes in terms of specific surface area, pore volume, pore size, functionality, ion-exchange capacity, conductivity, and reactivity are denoted on the resulting nanocomposite. Generally, the surface area for carbonaceous materials shows a general decline with the degree of modification and/or functionalization, while the adsorption capacity is increased [112]. NMs, including metal NPs such as Fe, Cu, Au, Pt, Pd, Ru, Ag, Co, and Ni, are the subject of great scientific and economic interest for oil-refining, chemical-manufacturing, and environmental catalysis applications [96]. In adsorption processes for the removal of heavy metals from contaminated water, iron and iron oxide NPs, immobilized in carbonaceous matrixes, have been extensively described, such as Cd^2+^, Cu^2+^, Ni^2+^, Co^2+^, Cr^6+^, Pb^2+^, As^3+^, and Hg^2+^ [103,113]. Additionally, the inclusion of metal NPs, such as MgO, CaO, Fe_2_O_3_, Al_2_O_3_, Fe^0^, and Ag^0^, on carbon nanocomposites can successfully improve the ligand-binding density by increasing the positive charge, allowing the higher removal of inorganic compounds than raw carbon materials [101]. Adsorption processes with carbon-based nanocomposites are also applicable for contaminated soils and air-pollution treatment. In the case of GHGs, nanocomposites using cocoa shell with amino groups and immobilized cobalt NPs were reported to effectively increase the CO_2_ adsorption capacity from 0.015 to 0.24 mmol/g [114], or in the case of soil remediation, modified magnetic BC was promisingly applied in the immobilization of heavy metals for the remediation of a multi-contaminated soil [115].

### 5.2. Silica-Based Nanocomposites

Silica (SiO_2_) is one of the commonly used supports for NP immobilization. Santa Barbara amorphous-type material (SBA-15) and Mobil Composition of Matter (MCM-41) are the most common types of amorphous-ordered, mesoporous silicas used as support in catalytic applications due to their extraordinary textural properties. Tetraethyl orthosilicate (TEOS) is the most common precursor for these ordered mesoporous silicas by reacting it with a template made of micellar rods or using sol-gel methodology [96]. Moreover, synthesis through the Stöber method and reverse micro-emulsion method yields materials with a controllable size, shape, and structure. Silica-based materials have gained attention as a scaffold or matrix for different NMs due to their superior properties, such as chemical inertness, thermal stability, transparentness to light and magnetism, lower van der Waals interactions, water-solubility, nontoxicity, and biocompatibility, over their counterparts [116]. Additionally, these materials have a high adsorption capacity, low permeability, high chemical and mechanical stability, large surface area, and narrow pore-size distributions [117]. The aforementioned characteristics render silica composites suitable for optical, magnetic, electrical, biomedical, environmental, and catalytic applications [118]. Table 3 summarizes several reports about the use of NMs for the adsorption of different pollutants.

The insertion of NMs onto siliceous matrixes changes and increases the desired properties of the resultant nanocomposites, since their main functions are mostly determined by such NMs, but provides an inert, transparent, stable, and heterogenous support. The sufficiently large pore size of mesoporous silica and the high density of silanol groups allows the integration of large molecules, surface functionalization, and the introduction of a variety of NPs for their application in catalysis, ion exchange, sensing, and adsorption, where stereochemical configurations, charge densities, specific binding sites, and acidities are needed. In adsorption processes, the attachment of NPs or a specific functionality onto an inert high specific surface area creates active sites for the selective adsorption of the target species. Several metal NPs (Pd, Ag, Ni, etc.) have been successfully immobilized on silica materials, such as aerogels, spheres, emulsions, zeolites, aluminosilicates, and clays [131,132,133,134,135]. The use of silica-based NMs has been extended to the adsorption and separation of hazardous metal ions, radioactive nuclides, or minor actinides from wastewater and high-level radioactive waste [136]. Recent studies have reported the use of nano-copper supported on mesoporous silica for the adsorption of dibenzothiophene in fuel oils [137]. Furthermore, the immobilization of hydrophilic NPs, derived from carbon black, on silica beads to enhance the removal of Cd^2+^, Ni^2+^, and Pb^2+^ [129] has been revealed. Additionally, silica aerogel composites embedded with nano lanthanum hydroxide to promote the removal of phosphate [95] have recently been reported. Furthermore, the use of gold NPs supported on amido-amidoxime-functionalized silica for toluene-vapor adsorption [130] and the sequestration of heavy metal ions from aqueous solutions using biosynthesized silica-based zinc oxide nanocomposites with agricultural wastes as a source of silica [138] have been explored.

### 5.3. Polymer-Based Nanocomposites

Polymer nanocomposites consist of a polymer or a copolymer, filled with synthetic or natural inorganic compounds to improve their chemical and physical properties or to reduce costs by acting as a diluent for the polymer [139]. The resultant nanocomposites combine the inherent nanoparticle qualities with the stability, controlled pore size, and surface chemistry of the polymeric matrix, including the benefits provided for the mechanical, thermal, optical, and electronic properties of such host material [140]. Several polymers have been used as supporting materials, including chitosan, porous resins, alginate, gelatin, and cellulose, for the immobilization of a variety of NPs, such as metallic oxides, zero-valent metals, single-enzyme NPs, among others [141,142,143]. Additionally, some NMs, such as reduced GO nanosheets, have been used for the self-assembling of nanostructured polymer composites [144]. The variety and availability of polymer-based nanocomposites and the high interfacial activity of the nanofillers makes them suitable for environmental remediation, catalytic degradation, detection and sensing, and green chemistry, among other applications [145,146,147]. The enhanced removal efficiencies of numerous toxic and recalcitrant pollutants, such as dyes (indigo carmine, methyl orange, methylene blue, crystal violet, reactive black, malachite green), heavy metal ions (Cr^2+^, Pb^2+^, As, Co, Ni^2+^, Cu, V, Sr^2+^, Hg^2+^), and organic molecules (diclofenac, nitrobenzene, trichlorophenol) have been reported using adsorbent-based polymeric nanocomposites [127,128,140,146,148]. Moreover, the selective adsorption of some species in the presence of competitors has been successfully tested [149]. MOFs are a subclass of coordination polymers with inorganic nodes bonded by organic ligands building a 3D spongy structure [111]. As a new functional material that combines an ultra-high SSA (up to 5900 m^2^/g) with high modularity, varied functionality, and strong metal–ligand interactions [96,111], MOFs have a wide application prospect in adsorption, separation, and gas storage [126], as well as their suitability for supporting active NPs for environmental remediation and catalytic purposes [150,151].

## 6. Conclusions

This article provides an overview of the vibrant and increasing scientific interest that has been awoken in manufacturing engineered NMs, which have been immobilized in different matrixes, for environmental applications. These purposes include their use for producing renewable energies through biological and electrochemical systems, as well as the removal of priority contaminants and GHGs from industrial discharges by microbial and physicochemical processes. There certainly are several avenues of opportunities to expand the application of immobilized NMs for environmental processes, which deserve consideration in future works. For instance, efforts should be made towards optimizing the immobilization of NMs in different matrixes to prevent their detachment during environmental applications. The tailoring of nanocomposites, integrating the catalytic properties of distinct NMs, should also be explored for electrochemical and catalytic processes intended for removing recalcitrant contaminants and GHGs. Finally, strategies to regenerate NMs to promote their recycling during their applications are also demanded.

## Figures and Tables

**Table 1:** Application of immobilized nanomaterials for energy production.

Immobilized NMs	Application	Achievement	Reference
**Bioprocesses**			
MNPs	Methane production from low-strength wastewater	3.6-fold higher methane production as compared to the control bioreactor	[12]
BIO	Methanogenic process	42% higher production of methane as compared to control	[13]
GO-OX	Methanogenic process	Higher COD and ammonium removal from protein-rich wastewater as compared to control	[14]
MNPs	Fermentative production of ethanol	Ethanol productivity of 264 g/L-h from corn starch, maintained in several cycles	[22]
MNPs	Fermentative production of ethanol from *Sesbania aculeate* biomass	Immobilized cellulase produced 5.3 g/L of ethanol along with the reuse of the nano-biocatalysts several times	[23]
MNPs	Biodiesel production from algal oil	Yield reaching 94% was maintained several cycles under optimal conditions	[24]
**Electrochemical processes**			
Pd, Pt	Pd NPs immobilized together with different microorganisms to produce energy in BES	Enhanced production of hydrogen or electricity as compared to controls without NPs	[26,27,28,29]
Co_3_S_4_-graphene	Lithium battery	Better cycling and greater reversible capacity as compared to the pristine Co_3_S_4_ electrode	[32]
Ni(OH)_2_-graphene	Supercapacitor	Better electrode–electrolyte interaction, enhancing the conductivity of ions and electrons, resulting in a higher performance	[34]
Graphene-CX	Aqueous supercapacitor	Combination of high porosity and electrical conductivity for 25% more capacitance and 100% more power than undoped CX at high current densities	[35]
S-I Pd aerogel	Microfluidic fuel cell	First time microwave-assisted synthesis of Pd aerogels with enhanced power densities for formic acid electro-oxidation	[36]

BES, bio-electrochemical systems; BIO, biogenic iron oxide; COD, chemical oxygen demand; CX, carbon xerogel; GO-OX, graphene oxide-organic xerogel; MNPs, magnetic nanoparticles; NPs, nanoparticles; S-I, self-immobilized.

**Table 2:** Application of immobilized nanomaterials for the removal of recalcitrant pollutants.

Immobilized NMs	Removed/Transformed Contaminants	Achievement	Reference
**Bioprocesses**			
Pd in MEC	TCE	93% removal efficiency vs. only 48% in the absence of Pd	[41]
Pd in AGS	IOP	81% removal of IOP vs. only 61% in the absence of Pd	[37]
Pd in AGS	NP and Cr^6+^	Up to 20-fold higher conversion in Pd-amended system as compared to control without Pd	[39]
Pd in AGS	Azo dyes	Up to 10-fold increase in reduction rate as compared to control	[40]
GSC	NB	30–50% higher removal of NB in the presence of graphene as compared to control	[42]
MGONS	IOP	82% removal efficiency vs. only 51% in the absence of MGONS	[43]
**Electrochemical systems**			
Fe/Co in NPC	Organic pollutants	Removal of 91% within 60 min and mineralization efficiency of 90% within 240 min under neutral conditions, good stability and reusability, with low metal leaching	[55]
Fe_3_O_4_ in MOF	Organic pollutants in OLL	66.67% COD removal efficiency and degradation of heterocycles, phenols, and esters with increase in low-molecular-weight organics abundance	[56]
Cu-GOr in CF	DCF	100% DCF degradation within 1 h at 1 V constant biased potential	[57]
Fe_3_O_4_ in CCB	Dyes	Up to 2-fold increased degradation rate as compared to Fe_3_O_4_ only	[49]
**Photocatalytic applications**			
La/TiO_2_ in L	DMP	74.4% removal vs. only 60.1% in the absence of La and only 18.3% in the absence of La/TiO_2_	[66]
Mn-CCMN	Dyes	Development of an effective and reusable photocatalyst for industrial wastewater treatment	[58]
ZnO in AC	Dyes	Promoting removal effect when adsorption and solar photocatalysis are simultaneously used for dye removal	[63]

AC, activated carbon; AGS, anaerobic granular sludge; CCB, conductive carbon black; CCMN, chitosan conjugated magnetic nano-biocomposite; CF, carbon film; DCF, diclofenac; DMP, dimethyl phthalate: GSC, graphene-sludge composite; IOP, iopromide; L, lithium silicon powder; MEC, microbial electrolysis cell; MGONS, magnetic graphene oxide nano-sacs; MOF, metal organic framework; NB, nitrobenzene; NP, nitrophenol; NPC, nitrogen-doped porous carbon rods; OLL, old landfill leachate; GOR, reduced graphene oxide; TCE, tri-chloro-ethylene.

**Table 3:** Adsorption of pollutants using immobilized nanomaterials.

Supporting Material	Nanomaterial	Pollutants	Q (mg/g)		SSA (m^2^/g)		Reference
			Support	Composite	Support	Composite	
A-Chitosan	Ni^2+^	Pb^2+^	21.0	50.3	NA	NA	[119]
Bamboo BC	MgO	Phosphate	1.3	370	NA	399	[120]
Chitosan	Fe^0^	Bisphenol-A	NA	65.2	NA	NA	[121]
Chitosan	Bio-CuO	Congo red, Eriochrome black T	NA	119.7, 235.7	15.84	25.3	[122]
CNT	MgO	Hg^2+^	NA	58.8	91.3	110.4	[90]
Corn stem BC	Fe-Mn	As^2+^	2.9	8.3	60.9	208.6	[123]
Douglas fir BC	α-Fe_2_O_3_, Fe_3_O_4_	Nitrate, fluoride	NA	15.5, 9.0	663	494	[124]
HCS BC	Fe^0^	Pb^2+^, Cu^2+^, Zn^2+^	NA	195.1, 161.9, 109.7	1216	603.4	[125]
MOF-WC	Co	Congo red, Methylene blue	613.6, 469.5	1117.0, 805.1	NA	170.4	[126]
Polydopamine	Graphene and Fe_3_O_4_	Methylene blue	110	140.3	NA	85.6	[127]
PC	Co	Sr^2+^	NA	3.21	NA	NA	[128]
Silica	HC	Cd^2+^, Pb^2+^, Ni^2^	NA	0.5, 8.9, 13.5	195	197	[129]
Silica	Au	Toluene	NA	1360	465.3	367.4	[130]
Silica aerogel	La(OH)_3_	Phosphate	19.2	153.8	561.0	252.7	[95]

A, aminophosphorylated; BC, biochar; CNT, carbon nanotubes carbon; HC, hydrophilic carbon; HCS, hydrophilic corn stalk; MOF-WC, metal–organic-framework wood composite; NA, not available; PC, phosphorylated chitosan.

## Data Availability

Not applicable.
